# Longitudinal Associations between Healthy Eating Habits, Resilience, Insomnia, and Internet Addiction in Chinese College Students: A Cross-Lagged Panel Analysis

**DOI:** 10.3390/nu16152470

**Published:** 2024-07-30

**Authors:** Liqing Yao, Kaixin Liang, Liuyue Huang, Jialin Xiao, Kaiji Zhou, Sitong Chen, Xinli Chi

**Affiliations:** 1 School of Psychology, Shenzhen University, Shenzhen 518060, China; 2 Faculty of Medicine, Medical Sciences Division, Macau University of Science and Technology, Macau 999078, China; 3 Department of Maternal and Child Health, School of Public Health, Sun Yat-sen University, Guangzhou 510080, China; 4 Department of Psychology, Faculty of Social Sciences, University of Macau, Macau 999078, China; 5 Department of Psychology, Faculty of Social Sciences, Lingnan University, Hong Kong 999077, China; 6 Department of Applied Social Sciences, The Hong Kong Polytechnic University, Hong Kong 999077, China; 7 Institute for Health and Sport, Victoria University, Melbourne 8001, Australia; 8 The Shenzhen Humanities & Social Sciences Key Research Bases of the Center for Mental Health, Shenzhen University, Shenzhen 518060, China

**Keywords:** healthy eating habits, resilience, insomnia, Internet addiction, college students, cross-lagged panel analysis

## Abstract

This study aimed to explore the longitudinal associations between healthy eating habits, resilience, insomnia, and Internet addiction by using a cross-lagged panel analysis of Chinese college students. Overall, 807 Chinese college students completed questionnaires on healthy eating habits, resilience, insomnia, and Internet addiction from August 2020 (time 1, T1) to November 2020 (time 2, T2), and were selected for the data analyses. Healthy eating habits (T1) had significant effects on resilience (T2; β = 0.064, *p* < 0.05) and insomnia (T2; β = −0.064, *p* < 0.05), but not Internet addiction (T2; β = −0.028, *p* > 0.05). Insomnia (T1) negatively predicted resilience (T2; β = −0.098, *p* < 0.01). Insomnia was bidirectionally associated with Internet addiction (Internet addiction at T1 to insomnia at T2: β = 0.085, *p* < 0.01; insomnia at T1 to Internet addiction at T2: β = 0.070, *p* < 0.05). Additionally, Internet addiction (T1) significantly predicted resilience (T2; β = −0.075, *p* < 0.05). This study further expanded the understanding of the longitudinal associations between healthy eating habits, resilience, insomnia, and Internet addiction, which provided higher-level evidence and important implications for the interventions for reducing college students’ Internet addiction, developing healthy eating habits, and improving resilience and sleep health.

## 1. Introduction

The Internet has become a more and more important aspect of human daily life due to its convenience, accessibility, and powerful functions in the background of the rapid development of science and technology [1,2]. In China, by June 2022, the number of Internet users had reached 1.05 billion with an Internet penetration rate of 74.4% [3]. However, in the process of experiencing the convenience brought by the Internet, negative effects cannot be ignored, for example, Internet addiction, which has become a strongly demonstrated, worrisome situation and attracted academic attention. Internet addiction is a form of problematic or pathological Internet use, which refers to periodic or chronic obsessions that are caused by the repeated use of the Internet, and frequently accompanied by some psychological symptoms (e.g., increased tolerance and withdrawal reactions) that are related to the addictive disorder [4,5]. In China, Internet addiction was relatively common among youths, especially college students, because they have more free time, lower self-control, and increased identity and lifestyle needs that they use the Internet to meet (e.g., online social interaction, shopping, learning, and gaming) [1,6]. A meta-analysis showed that the detection rate of Internet addiction among Chinese college students was 11% [7], which was higher than in Japan (3.7%) [8] and Italy (4.3%) [9]. Furthermore, previous studies have indicated that college students with Internet addiction have a higher risk of fatigue [10], higher severities of depression and anxiety symptoms [11,12], and stronger suicidal ideation [13]. Considering the association between Internet addiction and the multiple adverse outcomes in adolescents and young adults, it is essential to pay attention to the potential factors of Internet addiction, together with the further impacts caused by Internet addiction in these population groups.

Accumulating evidence has demonstrated that unhealthy lifestyle behaviors, such as unhealthy eating habits, were correlated with addictive disorders such as Internet addiction [14,15]. For instance, research involving Korean adolescents confirmed that high-risk Internet users had more irregular dietary behavior due to a loss of appetite, and higher frequencies of skipping meals and snacking, which might cause imbalances in intaking nutrients [16]. Another study from Turkey found that the prevalence of Internet addiction in college students with an eating disorder was significantly higher than those without an eating disorder [17]. Based on the studies above, a bidirectional relationship between eating habits and Internet addiction may exist in adolescents or young adults, which can prompt researchers to perform longitudinal studies to further confirm this. Exploring the bidirectional association might be beneficial to obtain more explicit pathways linking eating habits and Internet addiction.

Resilience, one of the most essential determinants of an individual’s mental health, may be a major influencing factor for healthy eating habits and Internet addiction. Resilience was defined as an individual’s ability to positively cope with life’s adversities [18,19], which could be an important target of treatment for anxiety, depression, and stress reactions [18]. Meanwhile, as a positive psychological resource and even a dynamic process, resilience is seen to be influenced by multiple environmental and behavioral factors [19,20]. Adopting healthy lifestyle practices, such as cultivating nutritious dietary habits, can augment an individual’s internal psychological reserves, consequently bolstering their ability to navigate and overcome stressors and challenges. Previous studies with cross-sectional designs have shown that resilience is significantly positively related to healthy eating habits [21,22]. However, these cross-sectional studies were unable to determine the specific direction of the relationship between resilience and healthy eating habits. The evidence resulting from longitudinal research, which may reflect that their relationship is bidirectional, is still lacking. Additionally, resilience plays an essential role in the development of Internet addiction. A meta-analysis supported that resilience was linked to problematic Internet use, and that it may help to reduce the risk of Internet addiction [23]. However, given the cross-sectional design of most of the included studies, they were not sufficient to establish the directionality between resilience and Internet addiction, hence it would be essential to perform longitudinal research to further confirm the directions of effects and to measure their evolution over time.

In addition to resilience, there may be other factors associated with healthy eating habits and Internet addiction, for example insomnia, one of the most usual psychosomatic factors. As a significant public health concern among college students, insomnia is a heterogeneous complaint reflecting reduced quality, duration, or frequency of sleep [24]. Accumulating research has revealed a strong link between insomnia and eating habits, which pointed out that insomnia may lead to an overconsumption of energy or late-night eating, which may lead to obesity [25]. Additionally, a cross-sectional study found that poor sleep quality was significantly associated with skipping breakfast, the consumption of energy drinks and sugar-sweetened beverages, and irregular eating among Japanese, middle-aged females [26]. Regarding the relationship between insomnia and Internet addiction, a longitudinal study in Taiwan indicated that students with Internet addiction had a higher chance of experiencing insomnia symptoms [27]. However, longitudinal studies on the effects of Internet addiction on insomnia still remain limited. Unhealthy behaviors, including unhealthy eating habits and Internet addiction, may be processed by the brain’s coping mechanisms and hedonic stimuli [28,29]. Therefore, developing unhealthy eating habits and excessively using the Internet while in a state of insomnia are performed to compensate for sleep disorders and to improve mood [28,29,30]. While insomnia is linked with healthy eating habits, it is also significantly associated with resilience. Two studies of Chinese and Japanese young people both reported that shorter sleep durations, irregular bedtimes, a greater severity of sleep disturbances, and higher levels of insomnia symptoms reduced their level of resilience [31,32]. Some neurological studies also suggested that insomnia symptoms may cause over-reactivity in a person’s amygdala, affecting the ability to withstand or recover from stressors [31,33]. However, despite previous studies indicating a potential influence of insomnia symptoms on resilience, the cross-sectional nature of these studies necessitates further longitudinal investigations to establish more specific causal relationships between these two variables.

To our knowledge, few previous studies have longitudinally investigated the bidirectional relationships between healthy eating habits, resilience, insomnia, and Internet addiction with pooled analyses among Chinese college students, as the existing studies have mainly conducted simple correlation analyses of two or three of these four factors. Hence, it is important to fill this gap in the knowledge and to supply related evidence in order to make a precise determination on public health interventions in the appropriate fields. Additionally, according to the biopsychosocial model, biological, psychological, and social factors present an interrelated relationship [34]. This model might support that healthy eating habits might be associated with psychological factors, including resilience and insomnia, via physiological mechanisms (e.g., a high fruit and vegetable consumption is conducive to increasing the levels of the factor that is related to brain-derived neurotrophins, which is beneficial for individuals to have good mental health [35]; and the lower levels of psychological disorders might be associated with lower risks of social behavioral problems, including Internet addiction [35]). Based on the theory and the previous studies above, in summary, this study aimed to explore the longitudinal associations between healthy eating habits, resilience, insomnia, and Internet addiction from the perspective of a cross-lagged panel analysis of Chinese college students.

## 2. Materials and Methods

### 2.1. Participants and Procedures

This longitudinal study was performed from August (time 1, T1) to November (time 2, T2) 2020 in China, and a cluster sampling method was utilized to recruit college students. The inclusion criteria for the students in the present study were as follows: (1) is aged ≥ 18 years old; (2) is not suffering from major diseases (e.g., cancer). The exclusion criteria were as follows: (1) has visual or hearing impairments; (2) has been suspended from, withdrawn from, or dropped out of school (including those at the stage of processing the relevant procedures); (3) has schizophrenia or other severe psychotic disorders. Before conducting the surveys, the sample size was calculated based on Gorsuch’s criterion, that is, the sample size should be ensured that the ratio of the number of scale items to the respondents is more than 1:5 [36]. The participants were recruited from Chinese social media (e.g., QQ and WeChat), and were invited to fill in the survey questionnaires via a Chinese online survey platform (Internet address: https://www.wjx.cn). Specifically, eating habits, resilience, insomnia, and Internet addiction were all measured at both T1 and T2. Participants were also asked to report their demographic information as covariates, including their age, gender, and body mass index (BMI). All of the items of the questionnaire were required to be answered before submission. All of the participants signed the electronic informed consent before filling in the questionnaires. The Human Research Ethics Committee of the Shenzhen University has approved this research project.

### 2.2. Measurements

#### 2.2.1. Internet Addiction

The Chinese version of Young’s 10-item Internet Addiction Test (IAT) was used to measure the severity of the Internet addiction and it presented good reliability and validity [37]. The scale contained 10 items, for example, “In order to get satisfaction, is it necessary for you to spend increasing time in using the Internet?” According to their experience, on this 2-point Likert scale, the participants could select the options of “0 = No” or “1 = Yes”. Total scores ranged from 0 to 10, and higher total scores showed a high level of IA symptoms. A total score of ≥4 indicated the likely presence of being addicted to the Internet. In this study, the Cronbach’s α values were 0.818 and 0.817 at T1 and T2, respectively.

#### 2.2.2. Healthy Eating Habits

Healthy eating habits were assessed by three items from the nutrient sub-scale of the Health Promoting Lifestyle Profile-II (HPLP-II) (Chinese version): i.e., “Daily fruit intakes”, “Daily vegetable intakes”, and “Daily breakfast intakes” [38]. Participants reported their frequency of fruit, vegetable, and breakfast intake according to a 4-point Likert scale (from 1 = Never to 4 = Routinely). Higher scores indicated a higher frequency of healthy eating habits, and the total scores were from 3 to 12. The HPLP-II was validated among Chinese youths [39]. In this study, the Cronbach’s α coefficients for the HPLP-II were 0.746 and 0.748 at T1 and T2, respectively.

#### 2.2.3. Resilience

Psychological resilience was measured by the Chinese version of the Connor-Davidson Resilience Scale (CD-RISC-10), which includes 10 items. The scale is a 5-point Likert scale (from 0 = never to 4 = almost always) with possible total scores ranging from 0 to 40 [40,41]. A higher total score indicated higher levels of resilience. The CD-RISC-10 has presented good reliability and validity in the Chinese population [40]. The Cronbach’s α coefficients for this scale in this study were 0.935 at both T1 and T2.

#### 2.2.4. Insomnia

The Youth Self-Rating Insomnia Scale (YSIS), Chinese version, was used to measure the levels of participants’ insomnia symptoms [42], and good validity and reliability of the scale were reported. It is a 5-point Likert scale with 8 items; participants were asked to report their feelings in the past month (e.g., “Do your sleep problems affect your daytime function?”). The total scores of this scale ranged from 8 to 40, and higher total scores indicated a high level of insomnia symptoms. The severity of the insomnia symptoms was defined as normal (<22), mild (22–25), moderate (26–29), or severe (≥30). In this study, the Cronbach’s α coefficients for this scale were 0.861 and 0.863 at T1 and T2, respectively.

### 2.3. Statistical Analysis

A common method bias test, descriptive statistics, and other preliminary analyses were conducted in SPSS 26.0 (IBM Corporation, Armonk, NY, USA). The cross-lagged analysis was performed using an Mplus 8.3. A maximum likelihood estimation was used to construct the cross-lagged model to explore the longitudinal associations between healthy eating habits, resilience, insomnia, and Internet addiction, based on the adjustments for students’ gender, age, BMI at T1, and the correlation of the four constructs in the same wave. In the descriptive statistics, the study classified four measured variables, such as exploring what percentage of participants had Internet addictive behaviors. The continuous indicators of these four measured variables were also used for the subsequent statistical analysis.

## 3. Results

### 3.1. Common Method Bias Test

Since all measurements were self-reported in this study, Harman’s one-way test [43] was conducted to examine the potential presence of a common method bias for all items at the two time points. The results of the analysis revealed that in both T1 and T2, six factors had eigenvalues greater than 1. The first factor that was extracted from the test accounted for 27.16% and 27.69% of the variance in T1 and T2, respectively. The variances of the largest factors were both below the widely accepted threshold of 40%, which showed that a significant common method bias did not exist in the current study.

### 3.2. Characteristics of the Participants

At baseline (T1), 1146 college students provided valid information. After matching the participants’ phone numbers with those at T1 and removing attrition data or extreme outliers, a total of 807 participants remained at follow-up (T2) and were selected in the final sample and data analyses. A chi-squared test and an independent *t*-test were utilized to examine the differences in the basic demographic information between students who dropped out and those who took part in the follow-up study (70.4%). The attrition analysis indicated that the participants who responded at both T1 and T2, when compared to the those who dropped out, were significantly more likely to be female (χ^2^ (1, n = 1146) = 6.17, *p* = 0.013); the results showed no differences with respect to age (*t* (1144) = 1.63, *p* = 0.103) or BMI (*t* (1144) = 0.16, *p* = 0.872). In the participants who responded in both waves, as for the characteristics related to the demographic information at baseline, the participants’ averaged age was 20.79 ± 1.76 years old. The percentage of male and female students was 32.5% and 67.5%, respectively. The averaged BMI was 20.19 ± 2.68 kg/m^2^ (underweight: 29.9%; normal weight: 60.3%; and overweight or obese: 9.8%). As for the measured variables of healthy eating habits, resilience, insomnia, and Internet addiction at both time points, it was found that the mean values for the healthy eating habit scores were 9.24 ± 1.87 and 8.89 ± 1.88 at T1 and T2, respectively. The mean scores for resilience were 24.88 ± 6.81 and 25.37 ± 6.93 at T1 and T2, respectively. The mean scores for insomnia symptoms were 18.30 ± 6.49 and 18.63 ± 6.35 at T1 and T2, respectively (T1: normal: 70.6%, mild: 14.0%, moderate: 10.7%, and severe: 4.7%. T2: normal: 68.2%, mild: 16.6%, moderate: 10.4%, and severe: 4.8%). Additionally, the mean scores for Internet addiction were 4.03 ± 2.84 and 3.42 ± 2.78 at T1 and T2, respectively; the prevalence rate was 52.0% and 44.7% at T1 and T2, respectively. The results of the descriptive statistics are presented in Table 1.

### 3.3. Preliminary Analyses

A Pearson correlation analysis was preliminarily used to investigate the associations between healthy eating habits, resilience, insomnia, and Internet addiction. The correlation analysis showed statistically significant correlations between these four variables at the two time points (see Table 2). A paired-sample *t*-test was used to compare the status of healthy eating habits, resilience, insomnia, and Internet addiction at T1 and T2 among the participants. The results showed that the level of healthy eating habits (*t* = 5.88, *p* < 0.001) and Internet addiction (*t* = 6.89, *p* < 0.001) had significantly decreased from T1 to T2. The level of resilience (*t* = −2.26, *p* < 0.05) had significantly increased. There was no significant change in the level of insomnia (see Table 3).

### 3.4. Cross-Lagged Analysis

**Figure 1:**
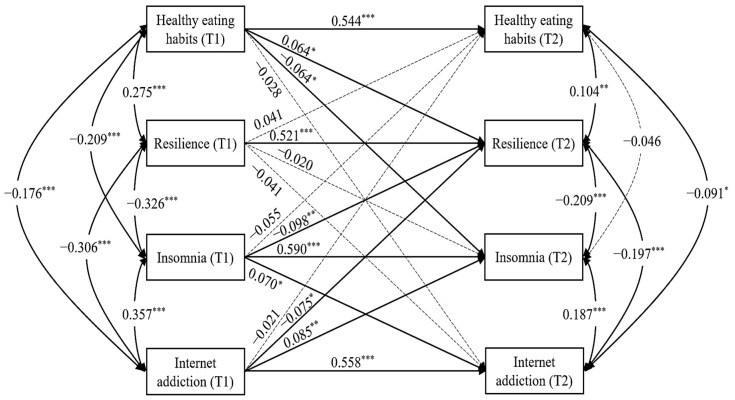
Cross-lagged relationships between healthy eating habits, resilience, insomnia, and Internet addiction. Note: T1, time 1; T2, time 2. Values are unstandardized coefficients. The model controlled for age, gender, and BMI. ^*^ *p* < 0.05, ^**^ *p* < 0.01, ^***^ *p* < 0.001.

A cross-lagged modeling approach to examine the association between healthy eating habits, resilience, insomnia, and Internet addiction was applied. The model fit statistics of this cross-lagged model were not reported since this model was a saturated model. After controlling for gender, age, BMI, and the correlation of the four constructs in the same wave, the results showed that healthy eating habits (T1) had significant effects on resilience (T2; β = 0.064, *p* < 0.05) and insomnia (T2; β = −0.064, *p* < 0.05), but not on Internet addiction (T2; β = −0.028, *p* > 0.05). Insomnia (T1) significantly negatively predicted resilience (T2; β = −0.098, *p* < 0.01). Insomnia was associated with Internet addiction bidirectionally, and the prediction effect of Internet addiction at T1 to insomnia at T2 (β = 0.085, *p* < 0.01) was higher than the predictive effect of insomnia at T1 to Internet addiction at T2 (β = 0.070, *p* < 0.05). Internet addiction (T1) significantly negatively predicted resilience (T2; β = −0.075, *p* < 0.05). Additionally, the model indicated that all the baseline variables predicted their corresponding follow-up variables significantly (*p* < 0.001). The details are presented in Figure 1 and Table 4.

## 4. Discussion

In the present study, a cross-lagged panel analysis was conducted to examine the relationships between healthy eating habits, resilience, insomnia, and Internet addiction in Chinese college students, utilizing two-wave data. A correlation analyses indicated significant correlations of these variables across these two waves, with notable declines observed in healthy eating habits and Internet addiction, and a notable increase observed in resilience in T2 relative to T1. Notably, the cross-lagged panel model results supported that a higher frequency of healthy eating habits predicted higher levels of resilience. Both higher levels of insomnia and Internet addiction predicted lower levels of resilience. The present study also found that a higher frequency of healthy eating habits predicted a lower risk of insomnia. Moreover, insomnia and Internet addiction were identified as interacting as causal factors in this study. The findings of this study offered innovative perspectives on the connections of causality between healthy eating habits, resilience, insomnia, and Internet addiction in college students. These findings could also provide scientific evidence for early interventions to develop healthy lifestyle behaviors, enhance mental health, and prevent Internet addiction among college students.

In the present study, there were prospective effects of healthy eating habits, insomnia, and Internet addiction on resilience. Firstly, consistent with the findings of a previous cross-sectional study [21], the mechanism of healthy eating as a predictor of enhanced resilience can be explained by the premise that individuals who keep a healthy diet generally intake abundant fruits and vegetables, and that various phytochemicals (e.g., carotenoids and polyphenols) possess biological functions that can promote the development of pressure resilience; specifically, phytochemicals and vitamins may play a key role in enhancing psychosocial stress coping and resilience through their effects of preventing oxidative stress and inflammation in the brain [21,44,45]. Moreover, as another kind of healthy eating habit, regular breakfast consumption can prevent the dysfunction of brain-derived neurotrophic factors and reduce the risk of depression, which can enhance mental resilience [46,47]. Secondly, this study provided evidence that a high severity of insomnia symptoms may predict low resilience, which can be explained by the premise that disturbed sleep may predispose a person to low resilience through sensitizing their attentiveness to negative emotional stimuli and attenuating their responsivity to positive events, which is in line with the stress-diathesis model [48,49,50]. Conversely, restoring sleep health appears to reduce the risks that are indicated by perceived stress, sleep responses, and rumination, thereby promoting more effective recovery from stressors and emotional dysregulation, thereby improving resilience [48]. Thirdly, the current study reported that Internet addiction predicted low levels of resilience, which was in line with the evidence of several previous studies [51,52]. It was explained that individuals with Internet addiction may lack social skills, which can make it difficult to establish good social relationships and lead to a lack of access to social support in the real world [53]. The social isolation can subsequently aggravate perceived stress and reduce resilience [52]. Therefore, healthy eating habits, insomnia, and Internet addiction have significant impacts on college students’ resilience, which emphasizes that healthy lifestyle behaviors are conducive to mental development. However, the prospective influences of resilience on the other variables were not observed in this study, which was inconsistent with other previous studies [54,55,56]. One possible reason is that most of the previous studies were cross-sectional designs and they might be unable to unequivocally formulate cause-and-effect or even bidirectional relations between resilience and the other factors. Hence, it is essential to perform more longitudinal studies to further examine the exact effects of resilience on those other variables and then examine the potential bidirectional associations.

In the present study, it was shown that a high frequency of healthy eating habits may be a protective factor against insomnia symptoms among college students, which was in line with studies focusing on the relationship between dietary patterns and insomnia [57,58]. These findings can be attributed to the fact that certain foods are abundant in nutrients that have sleep-enhancing characteristics [59,60]. For instance, certain vegetables (e.g., dark green, leafy vegetables) are rich in magnesium, which is a cofactor in neurotransmitter synthesis [60]. A double-blind placebo-controlled clinical trial verified that magnesium supplementation resulted in high melatonin secretion [61]. Melatonin can increase sleep propensity through thermoregulatory mechanisms, which is conducive to preventing insomnia symptoms [60,62]. Thus, based on the physiological factor pathway above, developing healthy eating habits and improving diet quality can be used to promote sleep health in college students.

Our findings showed that Internet addiction and insomnia had bidirectional associations, which was in accordance with the literature [27,63]. A fundamental transdiagnostic neurobiological and psychosocial framework indicates several pathways through which insufficient sleep might impact psychobehavioral disorders and through which psychobehavioral disorders may impact sleep quality [64]. Regarding Internet addiction as a predictor of insomnia, it was demonstrated that the excessive use of the Internet had negative effects on the sleep–wake rhythm of individuals, which could reinforce insomnia [27,65]. Additionally, as individuals with Internet addiction are reported to manage low levels of self-control skills frequently, problematic Internet use may prevent them from keeping a regular schedule, then lead to reduced sleep time or the exacerbation of insomnia symptoms [66,67]. As for the influence of insomnia on Internet addiction, one of the possible explanations is that participants who have difficulty in falling asleep during the night-time or remaining asleep may develop a higher severity of depressive symptoms, and they may be likely to engage in Internet use and develop Internet addictive behaviors [27,68,69]. Moreover, from the perspective of cognitive neuroscience, in line with recent studies, this finding can be explained by the premise that a wide range of abnormal connections in the hippocampus including memory, reward motivation, and cognitive control, especially the coupling between the posterior hippocampus (pHIP) and caudate nucleus reflecting poor sleep-reward interactions, might mediate sleep problems and Internet addiction, and sleep disorders may further aggravate the degree of Internet addiction and be mediated by this relationship [70,71]. In conclusion, exploring the bidirectional association and mechanism between insomnia and Internet addiction is beneficial for the subsequent development of bidirectional interventions.

Additionally, the results showed that all the baseline variables significantly predicted their corresponding follow-up variables. Firstly, college students with healthy eating habits at baseline may increase the overall dietary quality or maintain the high stability of healthy patterns, which was in line with the findings in prospective cohort studies among older adults [72,73]. Students with healthy eating habits generally actively enhance the acquisition of nutrition knowledge and as such an improvement may contribute to better dietary habits and quality [74]. An effective strategy of increasing the nutritional content of the curriculum may be constructed to help college students to achieve healthier dietary patterns. Secondly, regarding high resilience predicting subsequent higher resilience, according to resilience framework theory, individuals with positive psychological resilience can promote the reintegration of resilience, thus enabling them to reach greater resilience subsequently [75]. Thirdly, college students’ prior insomnia significantly predicted their later insomnia, and this stability was somewhat consistent with the results of a prospective cohort study in Norway [76]. Insomnia is usually consolidated or further escalated through maladaptive coping behaviors (e.g., daytime napping) and negative beliefs about sleep (e.g., exaggeration of the consequences of the lack of sleep) [77,78]. Lastly, our findings also supported that excessive Internet use is likely to continue in college students; that is, Internet addiction may be a relatively stable behavioral pattern, being somewhat in line with previous studies [79,80]. It is possible that once the pattern of pathological use of the Internet is established, it may not be easily changed, as with other addictive behaviors [79]. Hence, based on the findings of autoregression above, it is essential to examine more characteristics of those with a persistent healthy diet, resilience, insomnia, and Internet addiction in the future research.

### Strengths and Limitations

The present study used a combination of prospective design and structural equation modeling to comprehensively identify associations between healthy eating habits, resilience, insomnia, and Internet addiction among college students. This longitudinal study contributes a better understanding of the directionality of these factors by cross-lagged panel model, such as the influence of healthy eating habits on resilience and insomnia, the impacts of insomnia and Internet addiction on resilience, and the bidirectional relationship between insomnia and Internet addiction, thereby promoting causal inference and appealing for more attention to improving the primary care of lifestyle behaviors and mental health, and the prevention of Internet addiction in college students.

Several underlying limitations need to be acknowledged in the current study. First, the measurements of the factors were conducted by self-reported questionnaires, which may result in recall bias, although adequate reliability existed in the self-reported measurements. To make the results more accurate, the subjective indicators and objective ones can be considered to be combined in future studies. Second, although we have included age, gender, and BMI as covariates, other potential confounders (e.g., subjective socioeconomic status) that might impact the relations between healthy eating habits, resilience, insomnia, and Internet addiction were not considered in this study. Therefore, future research can further control other potential factors as well. Third, attrition data were handled by using the maximum likelihood method, which is widely regarded as the best available method [81]. However, it may not eliminate all limitations and bias. There were important significant differences in gender and healthy eating habits between T1 and T2. Therefore, our findings need to be interpreted cautiously. Fourth, we only measured the frequency of the dietary components of healthy eating habits with a lack of more detailed dietary materials such as the quantitative data and the analyses of other components, which could not rule out the potential confounding factors from the overall diet quality. Hence, more accurate and comprehensive dietary measurements, such as semi-quantitative food-frequency questionnaires, are recommended to obtain more precise information in future studies. Fifth, due to the short 3-month interval, it still remains unclear whether there will be any changes of the longitudinal associations over time between the four factors in the current study. It is necessary to perform long-term interval research to further determine the direction of the relationships between these variables. Lastly, since the cross-lagged panel analysis was conducted using only two waves of data, there might be no continuous time relationship between these variables in this study. Future studies need to use a cross-lagged panel model with three or more waves of data in order to properly explore the persistent bidirectional associations between the variables.

## 5. Conclusions

Generally, this longitudinal study using a cross-lagged analysis further expanded the understanding of the longitudinal associations between healthy eating habits, resilience, insomnia, and Internet addiction. Specifically, this study suggests that healthier eating habits may directly predict a higher level of resilience, and less severe symptoms of insomnia. A higher severity of insomnia may predict a lower level of resilience. Insomnia may be bidirectionally associated with Internet addiction. Additionally, Internet addiction may also significantly negatively predict resilience. This study provided higher-level evidence and important implications for the interventions aimed at reducing college students’ Internet addiction, together with the measures for developing their healthy eating habits and improving their resilience and sleep health.

## Figures and Tables

**Table 1:** Characteristics of the participants.

	M ± SD/N (%)
**Gender**	
Male	262 (32.5%)
Female	545 (67.5%)
**Age**	20.79 ± 1.76
**BMI**	20.19 ± 2.68
Underweight (<18.5 kg/m^2^)	241 (29.9%)
Normal weight (18.5–23.9 kg/m^2^)	487 (60.3%)
Overweight or obese (≥24 kg/m^2^)	79 (9.8%)
**Healthy eating habits (T1)**	9.24 ± 1.87
**Healthy eating habits (T2)**	8.89 ± 1.88
**Resilience (T1)**	24.88 ± 6.81
**Resilience (T2)**	25.37 ± 6.93
**Insomnia (T1)**	18.30 ± 6.49
Normal	570 (70.6%)
Mild	113 (14.0%)
Moderate	86 (10.7%)
Severe	38 (4.7%)
**Insomnia (T2)**	18.63 ± 6.35
Normal	550 (68.2%)
Mild	134 (16.6%)
Moderate	84 (10.4%)
Severe	39 (4.8%)
**Internet addiction (T1)**	4.03 ± 2.84
No	387 (48.0%)
Yes	420 (52.0%)
**Internet addiction (T2)**	3.42 ± 2.78
No	446 (55.3%)
Yes	361 (44.7%)

Note: M, mean; SD, standard deviation.

**Table 2:** Pearson correlation analysis of healthy eating habits, resilience, insomnia, and Internet addiction.

Variables	1	2	3	4	5	6	7	8
1. Healthy eating habits (T1)	1							
2. Healthy eating habits (T2)	0.580 ^***^	1						
3. Resilience (T1)	0.263 ^***^	0.197 ^***^	1					
4. Resilience (T2)	0.228 ^***^	0.230 ^***^	0.591 ^***^	1				
5. Insomnia (T1)	−0.201 ^***^	−0.175 ^***^	−0.336 ^***^	−0.309 ^***^	1			
6. Insomnia (T2)	−0.203 ^***^	−0.184 ^***^	−0.265 ^***^	−0.358 ^***^	0.643 ^***^	1		
7. Internet addiction (T1)	−0.172 ^***^	−0.144 ^***^	−0.307 ^***^	−0.282 ^***^	0.356 ^***^	0.314 ^***^	1	
8. Internet addiction (T2)	−0.146 ^***^	−0.172 ^***^	−0.246 ^***^	−0.336 ^***^	0.289 ^***^	0.349 ^***^	0.607 ^***^	1

Note: T1, time 1; T2 time 2. ^***^ *p* < 0.001.

**Table 3:** The changes in the measured variables from T1 to T2.

Measure Variables	Time	M	SD	95% CI		*t*
				LL	UL	
Healthy eating habits	T1	9.24	1.87	0.24	0.47	5.88 ^***^
	T2	8.89	1.88			
Resilience	T1	24.88	6.81	−0.92	−0.07	−2.26 ^*^
	T2	25.37	6.93			
Insomnia	T1	18.30	6.49	−0.71	0.04	−1.74
	T2	18.63	6.35			
Internet addiction	T1	4.03	2.84	0.43	0.78	6.89 ^***^
	T2	3.42	2.78			

Note: M, Mean; SD, standard deviation; T1, time 1; T2 time 2. ^*^ *p* < 0.05, ^***^ *p* < 0.001.

**Table 4:** Cross-lagged model of measure variables.

Path	β	SE	LLCI	ULCI
Healthy eating habits (T1) → Healthy eating habits (T2)	0.544 ^***^	0.027	0.491	0.597
Healthy eating habits (T1) → Resilience (T2)	0.064 ^*^	0.030	0.006	0.122
Healthy eating habits (T1) → Insomnia (T2)	−0.064 ^*^	0.029	−0.121	−0.007
Healthy eating habits (T1) → Internet addiction (T2)	−0.028	0.029	−0.084	0.028
Resilience (T1) → Resilience (T2)	0.521 ^***^	0.038	0.447	0.596
Resilience (T1) → Healthy eating habits (T2)	0.041	0.034	−0.026	0.108
Resilience (T1) → Insomnia (T2)	−0.020	0.030	−0.080	0.040
Resilience (T1) → Internet addiction (T2)	−0.041	0.037	−0.114	0.032
Insomnia (T1) → Insomnia (T2)	0.590 ^***^	0.029	0.532	0.647
Insomnia (T1) → Healthy eating habits (T2)	−0.055	0.033	−0.119	0.010
Insomnia (T1) → Resilience (T2)	−0.098 ^**^	0.035	−0.166	−0.030
Insomnia (T1) → Internet addiction (T2)	0.070 ^*^	0.034	0.002	0.137
Internet addiction (T1) → Internet addiction (T2)	0.558 ^***^	0.032	0.496	0.621
Internet addiction (T1) → Healthy eating habits (T2)	−0.021	0.034	−0.088	0.047
Internet addiction (T1) → Resilience (T2)	−0.075 ^*^	0.032	−0.138	−0.012
Internet addiction (T1) → Insomnia (T2)	0.085 ^**^	0.029	0.028	0.142

Note: T1, time 1; T2, time 2; SE, standard error; LLCI, lower limit of the 95% confidence interval; ULCI, upper limit of the 95% confidence interval. ^*^ *p* < 0.05, ^**^ *p* < 0.01, ^***^ *p* < 0.001.

## Data Availability

The data analyzed in the current study are available from the corresponding author on reasonable request.
